# Powder Metallurgy Synthesis of Heusler Alloys: Effects of Process Parameters

**DOI:** 10.3390/ma12101596

**Published:** 2019-05-15

**Authors:** Riaz Ahamed, Reza Ghomashchi, Zonghan Xie, Lei Chen

**Affiliations:** School of Mechanical Engineering, University of Adelaide, Adelaide, SA 5005, Australia; reza.ghomashchi@adelaide.edu.au (R.G.); zonghan.xie@adelaide.edu.au (Z.X.); lei.chen@adelaide.edu.au (L.C.)

**Keywords:** Heusler alloy, powder processing, process parameters, microstructure, martensite

## Abstract

Ni_45_Co_5_Mn_40_Sn_10_ Heusler alloy was fabricated with elemental powders, using a powder processing route of press and sinter, in place of vacuum induction melting or arc melting route. The effects of process parameters, such as compaction load, sintering time, and temperature, on the transformation characteristics and microstructures of the alloy were investigated. While the effect of compaction pressure was not significant, those of sintering time and temperature are important in causing or annulling martensitic transformation, which is characteristic of Heusler alloys. The processing condition of 1050 °C/24 h was identified to be favorable in producing ferromagnetic Heusler alloy. Longer durations of sintering resulted in an increased γ-phase fraction, which acts as an impediment to the structural transformation.

## 1. Introduction

Ni–Mn–X (X – Ga, Sn, In, Sb)-based ferromagnetic Heusler alloys are multifunctional materials on account of their multiferroic nature [1]. This has its origin in a couple of remarkable and reversible solid-state transformations, viz., the primary martensitic phase transformation and the secondary magnetic transition. While the former transforms the parent cubic *L*2_1_ austenite to modulated orthorhombic/non-modulated tetragonal martensite, the latter changes the magnetic order of the phases from ferromagnetic austenite to anti-ferromagnetic/paramagnetic martensite [2]. The reversibility of the transformations is uniquely identified with a shape memory effect (SME), either magnetic (MSME) or metamagnetic (MMSME). In the former, strain recovery is by martensitic twin variants reorientation under an applied field as in Ni–Mn–Ga alloys [3], while in the latter, it is by a field-induced reverse transformation from the martensite phase back to parent austenite phase, as in other Ga-free alloys (e.g., Ni_50_Mn_34_In_16_) [3]. MSME [4] and other effects of magnetocaloric [5,6,7,8], magneto-resistance [9,10], exchange bias [11], and direct conversion of heat into electricity [12] are interconnected [2], which explains the multifunctional behavior. 

These alloys are usually synthesized by liquid processing, followed by annealing. Examples of Heusler alloys prepared by arc/induction melting and characterized for their remarkable magnetostructural properties include compositions in the Ni_50_Mn_50-x_Sn_x_ system (composition rewritten) with large inverse entropy change, leading to a large inverse magnetocaloric effect [6], Ni_41_Co_9_Mn_40_Sn_10_ with large magnetic entropy change and magnetoresistance [5], compositions in the Ni_50_Mn_50-y_X_y_ (X = In, Sn, Sb) system with magnetic shape memory [13], Ni_43_Co_7_Mn_39_Sn_11_ with magnetic field-induced shape recovery by reverse phase transformation [14], and Ni_45_Co_5_Mn_40_Sn_10_ for potential energy conversion [15]. Other alloys, such as Ni_50_Mn_37_Sn_13_ [16], compositions in the Ni_50_Mn_50-x_Sn_x_ system (composition rewritten) [17], Ni_42_Co_8_Mn_39_Sn_11_ [18], compositions in the Ni_50_Mn_50-x_Sn_x_ system [19], and Ni–Co–Mn–Sn alloy [20] synthesized by liquid processing, deal with microstructural aspects, such as the effect of compositional variations on microstructure in response to heat treatments, crystal structures of austenite and martensite, determination of crystallographic phases, and stability of different crystallographic structures under varying temperatures and solidification microstructures respectively. Carried out under controlled conditions of a vacuum (10^−4^ bar), an inert atmosphere (argon) with/without the addition of suitable oxygen getter materials, such as Ta or Ti to prevent oxidation [21], arc/melting ensures compositional homogeneity, resulting in a stable single phase *L*2_1_ structure. Melt-spinning [22,23,24,25,26] produces highly textured samples with controlled austenitic grain size, suitable for practical applications. In a process in which the alloy melt stream is allowed to solidify rapidly on a fast-rotating (10 m/s to 60 m/s) substrate wheel, the wheel speeds which are an indication of the solidified foil thickness and its solidification rate, influence the magnetostructural phase transformations and characteristics of the alloys [27]. Directional solidification results in chemical segregation or a composition gradient with a control over the transformation temperatures. Bridgman–Stockbarger [28] and Czochralski [29] techniques of directional solidification have been used for preparing single crystals of the alloys. 

Solid processing by conventional powder metallurgy (P/M) has been used in a limited manner with alloy powders [30,31,32,33] with properties identical to or less than the bulk material. The use of elemental powders in a conventional compaction/sintering procedure has been attempted in the synthesis of a quinary Ni–Co–Mn–Sn–Cu alloy [34]. The transformational characteristics and the observed microstructure of the sintered quinary alloy are similar to cast alloy, thereby offering scope for further investigation. Given that good compositional control can be obtained with the use of elemental powders, the P/M route warrants further study to fabricate high performance Heusler alloys at low costs. This paper endeavors to elucidate the effects of the conventional powder metallurgy parameters, such as compaction load, sintering time, and temperature, upon the transformation characteristics and microstructural features of a quaternary Ni_45_Co_5_Mn_40_Sn_10_ Heusler alloy.

## 2. Materials and Methods

Quaternary Ni_45_Mn_40_Co_5_Sn_10_ alloys were prepared using the P/M technique from commercial purity elemental powders of nickel, manganese, cobalt, and tin. The particle size and distribution were measured on a Mastersizer 2000 (Malvern Panalytical, Worcestershire, UK) laser diffraction particle size analyzer. D (v,0.5) sizes of the powders are tabulated in Table 1. D (v,0.5) refers to the size at which 50% of the sizes are smaller and 50% are larger. 

The individual powders were carefully weighed to atomic composition. About 0.5 to 1% A-wax (Acrawax; N, N’ Ethylene Bisstearamide) was added for lubrication. The powders were sealed in a clean cylindrical steel tube and mixed at 120 rpm for approximately 2 h with 5 mm diameter balls at an approximate ball to powder ratio of 4:1. They were then compacted into 11 mm diameter cylindrical compacts, using a tool steel die and punch on a Mohr and Federhaff universal testing machine with a maximum capacity of 200 kN. The green compacts were sintered in a high-temperature tube furnace (70 mm ID and a heating zone of 150 mm) by sealing them off inside silica tubes at argon-partial pressure at a heating rate of 5 °C/min. Table 2 shows the prepared samples and corresponding processing conditions. All samples were furnace cooled (furnace turned off after desired sintering time) to room temperature. 

The densities of the green and sintered compacts were determined in accordance with the Archimedes principle, using a custom-built density measuring apparatus. Phase transformation temperatures were determined through differential scanning calorimetry (DSC), using a TGA/DSC 2 equipment (Mettler-Toledo AG, Schwerzenbach, Switzerland) differential scanning calorimeter within the temperature range of 25–400 °C. Heating and cooling rates of 5 °C/min were followed in the heating and cooling routines. The enthalpy changes (ΔH) around the phase transformation were calculated from DSC data, using the ‘STARe’ software (Version 9.30, Mettler-Toledo AG, Schwerzenbach, Switzerland) associated with the equipment. Metallographic procedures were similar to those applied on the quinary alloy samples described elsewhere. A LECO LM 700 AT Microhardness Tester (LECO, Saint Joseph, MI, USA) with Vickers indenter with an applied load of 500 g for 15 s dwell time was used for microhardness measurements. Examination of microstructures was carried out on an Environmental SEM (Quanta FEG 450, Hillsboro, OR, USA) scanning electron microscope, equipped with an energy dispersive spectroscopy (EDS) attachment for composition determination. A Rigaku MiniFlex 600 X-ray diffractometer (XRD) (Rigaku, Akishima-shi, Tokyo, Japan) was used for phase determination. All the patterns were obtained using Cu-Kα radiation with a wavelength of 1.5406 Å. Additionally, XRD and M–H curves of the samples were obtained from another laboratory for the purpose of verification of our results.

## 3. Results and Discussion

The actual compositions of the samples were measured using EDS and listed in Table 2. The compositions are not too deviant from the starting composition of Ni_45_Co_5_Mn_40_Sn_10_, except for slight variations in Sn and Mn. This confirms that the powder metallurgy route can also be employed to synthesize Heusler alloys. 

### 3.1. Density and Hardness

**Figure 1:**
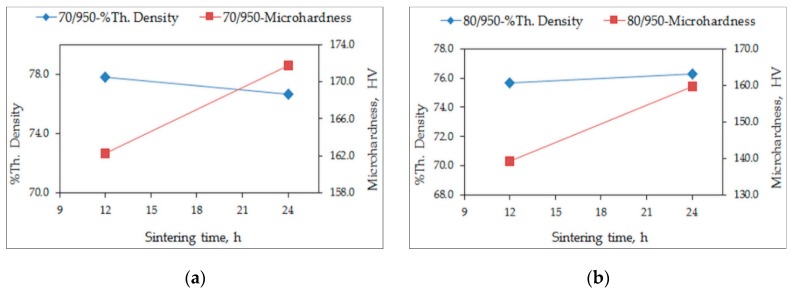
Variation of % theoretical density and microhardness with sintering time of: (**a**) S950a1, S950a2; (**b**) S950b1, S950b2.

**Figure 2:**
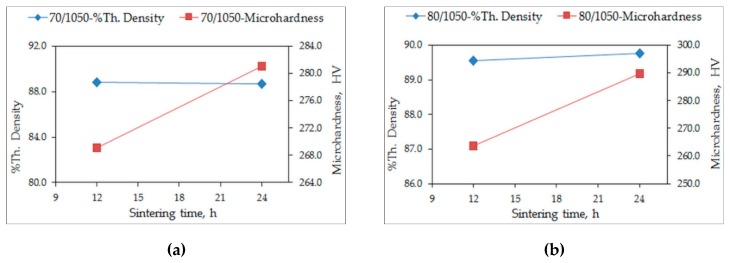
Variation of % theoretical density and microhardness with sintering time of: (**a**) S1050a1, S1050a2 and (**b**) S1050b1, S1050b2.

**Figure 3:**
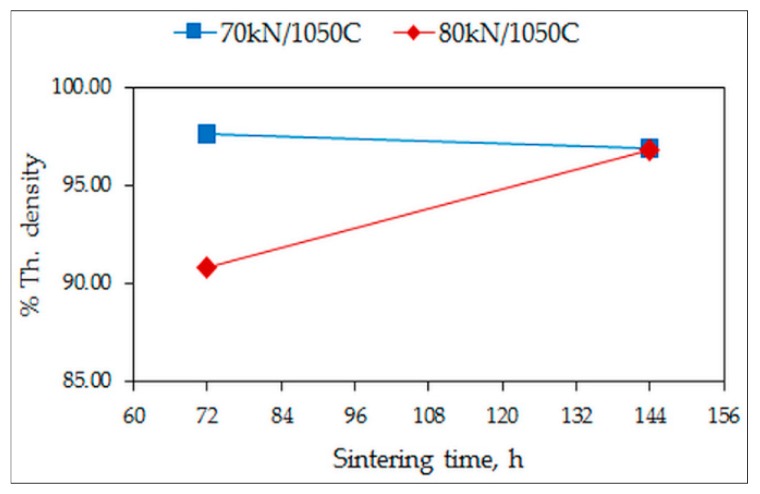
Variation of % theoretical density with sintering time of quaternary alloys S1050a3, S1050a4, S1050b3, and S1050b4.

The theoretical density of the quaternary alloy was determined as 8.02 g/cm^3^. The measured densities of the quaternary alloy samples are expressed as percentages of the theoretical density (% theoretical density). The % theoretical densities and the microhardness values of the conventionally sintered alloys, sintered for 12 h and 24 h, are plotted in Figure 1 and Figure 2. The variation in the % theoretical densities is around 77% for S950a1, S950a2, S950b1, and S950b2 alloys, as seen in Figure 1a,b, while it is around 89% for S1050a1, S1050a2, S1050b1, and S1050b2 alloys, shown in Figure 2a,b. The percentage of porosity of samples sintered at 950 °C is approximately 23%, while that of alloys sintered at 1050 °C is approximately 11%. Figure 2 shows that longer sintering times and higher temperatures are desirable for effective sintering and reduction in porosity. Microhardness variations of the alloys also reveal that specimens sintered at 1050 °C (S1050 alloys) have higher hardness values varying from 260 HV to 290 HV, while alloys sintered at 950 °C (S950 alloys) have relatively lower hardness values varying from 140 HV to 175 HV. In all of them, the microhardness is seen to be increasing with the sintering duration. The effect of compaction load is not as significant. The measured densities of the quaternary S1050a3, S1050a4, S1050b3, and S1050b4 alloys expressed as percentages of the theoretical density are plotted in Figure 3. After 144 h, the % density is approximately 96%.

**Figure 4:**
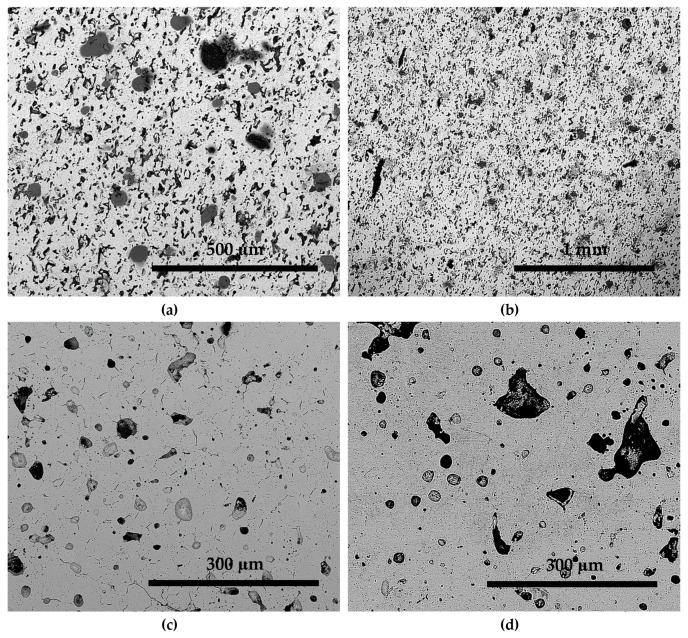
BSE images of samples: (**a**) S950a2; (**b**) S950b2; (**c**) S1050a2; and (**d**) S1050b2.

SEM micrographs of S950a2, S950b2, S1050a2, and S1050b2 alloys, sintered for 24 h, are shown in Figure 4. It can be seen that the alloys sintered at 950 °C show a poor microstructure with large amounts of porosity, as in Figure 4a,b. On the other hand, alloys sintered at 1050 °C show microstructures that are indicative of the occurrence of effective sintering in them, as in Figure 4c,d. Although more than one phase seemed to appear, the porosity is less in these alloys. The porosities of the alloys are in agreement with the measured densities discussed earlier. It becomes clear that higher temperatures are necessary for effective diffusion between powder particles. The effect of compaction is observed to be less significant. The compositions of the alloys listed in Table 2 are identical to the starting composition, with the Ni and Co values being nearly equal. However, alloys S950a1, S950a2, S950b1, and S950b2 have less Sn and more Mn. This may be attributed to the sintering temperature being insufficient for effective diffusion of the powder particles to attain homogeneity. As a result, the microstructures seen in Figure 4a,b show high levels of porosity. Alloys S1050a1, S1050a2, S1050b1, and S1050b2 also have less Sn and more Mn. However, the difference is about half the difference seen in S950a1, S950a2, S950b1, and S950b2 alloys. The sintering temperature and duration of sintering seemed to have favored increased diffusion, thereby reducing the inhomogeneity. This can be seen from the reduced porosity in the microstructures in Figure 4c,d and also from the measured densities.

**Figure 5:**
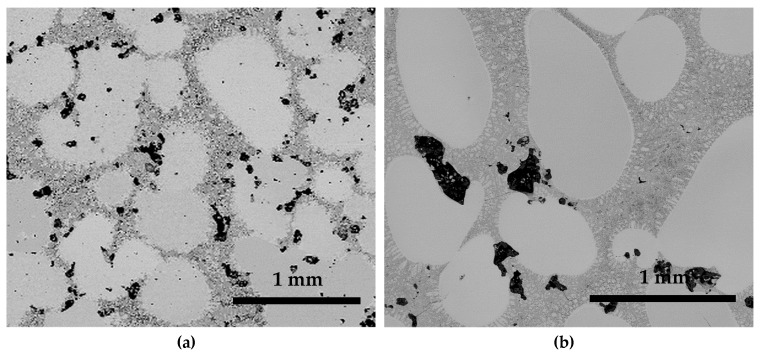
BSE images of samples: (**a**) S1050a3 and (**b**) S1050b4.

SEM micrographs of S1050a3 and S1050b4 alloys shown in Figure 5 suggest melting has occurred at an extended duration of sintering (72 and 144 h, respectively) resulting in a dendritic structure. The microstructures in Figure 5a,b show the light gray cells surrounded with a two phase mixture of light and dark gray regions, with porosity (black) being minimal. In Figure 5b of the sample sintered for 144 h, the progress of powder particle interaction is seen, with interparticle regions becoming more developed and the large light gray cells coarsened and smoothed off when compared to Figure 4a. The composition of S1050a3 is close to the starting composition with a small variation in Sn. However, when the sintering time increases to 144 h, the difference between Mn and Sn expands, which is an indication that more Sn has been removed from the powder mixture, S1050b4 and S1050a4. The reduction in the level of tin may be attributed to the tin moving into the pores, due to the capillarity effect, as Sn has a very low melting point (232 °C) and is expected to have very low viscosity at the sintering temperature.

### 3.2. Differential Scanning Microscopy

**Figure 6:**
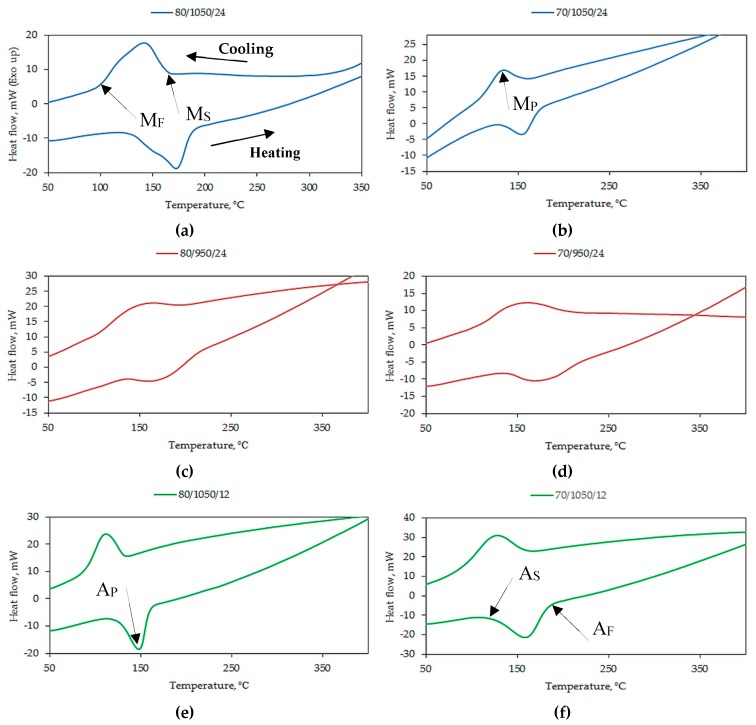

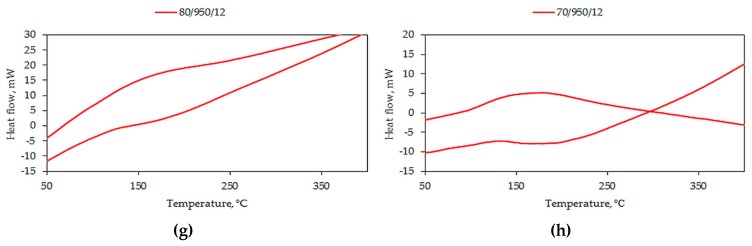
DSC curves of: (**a**) S1050b2; (**b**) S1050a2; (**c**) S950b2; (**d**) S950a2; (**e**) S1050b1; (**f**) S1050a1; (**g**) S950b1; and (**h**) S950a1.

Differential scanning calorimetry (DSC) measurements were obtained from identical heating and cooling cycles for all the samples. The austenite start (A_S_), finish (A_F_), and peak (A_P_), martensite start (M_S_), finish (M_F_), and peak (M_P_), martensitic transformation (A_S_ + M_F_)/2, thermal hysteresis (A_F_ − M_S_) temperatures, and enthalpy changes around phase transformation (ΔH) of the samples are presented in Table 3. These temperatures are also suitably identified in the DSC curves shown below in Figure 6. The enthalpy changes (ΔH) around the phase transformation are calculated from DSC data. 

Exothermic peaks corresponding to austenite–martensite transformation (cooling) and endothermic peaks corresponding to a reverse martensite–austenite transformation (heating) seen on the DSC curves indicate the occurrence of forward and reverse martensitic transformation, while the absence of peaks is not indicative of martensitic transformation. The effects of temperature and time on the phase transformations are to be examined. A sintering temperature of 950 °C resulted in an incomplete martensitic transformation in which the transformation and hysteresis temperatures could not be decisively determined. This can be seen in samples S950a1 and S950b1 in Figure 6h,g, respectively, wherein the sintering duration of 12 h also does not favor a complete transformation sequence. On the contrary, both forward and reverse martensitic transformation sequences are discerned in samples S950b2 and S9502 in Figure 6c,d, sintered at 950 °C for 24 h. The transformation and hysteresis temperatures determined for these samples appear to be an aberration as the peaks are not sharp and narrow. It is conjectured that even a longer sintering duration at an insufficient sintering temperature could result in an inhomogeneity or, more likely, the incomplete synthesis of phases with large changes in the stoichiometry. Martensitic transformation is dependent on the composition and thermal events associated with it increase in sharpness and intensity upon additional annealing [35]. Quenching after annealing completes the formation of martensite. It can be seen that fast heating/cooling freezes the calorimetric response of the samples in terms of both the transition temperature and enthalpies [36].

**Figure 7:**
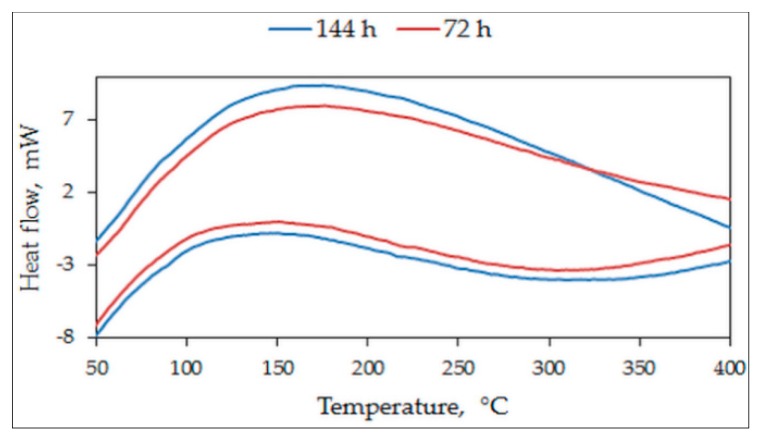
DSC curves of: Samples S1050a3 and S1050a4.

In samples S1050a1 and S1050b1, compacted at different pressures but sintered at 1050 °C and 12 h, the transformation temperatures are 103 °C and 107 °C, respectively. These are seen in Figure 6f,e. The temperatures are lower than 125 °C, obtained for the cast alloy [15]. In samples S1050b2 and S1050a2, seen in Figure 6a,b, the transformation temperatures are not very different from those reported in [12,15], because the sintering time for these samples was 24 h. Sintering temperature and duration of sintering are important. Figure 7 shows the DSC curves of samples S1050a3 and S1050a4. It can be seen that these samples record no martensitic transformation. The lack of martensitic transformation is due to the formation of other phases, which are explained in detail in the next section. 

### 3.3. Microstructure Analysis

**Figure 8:**
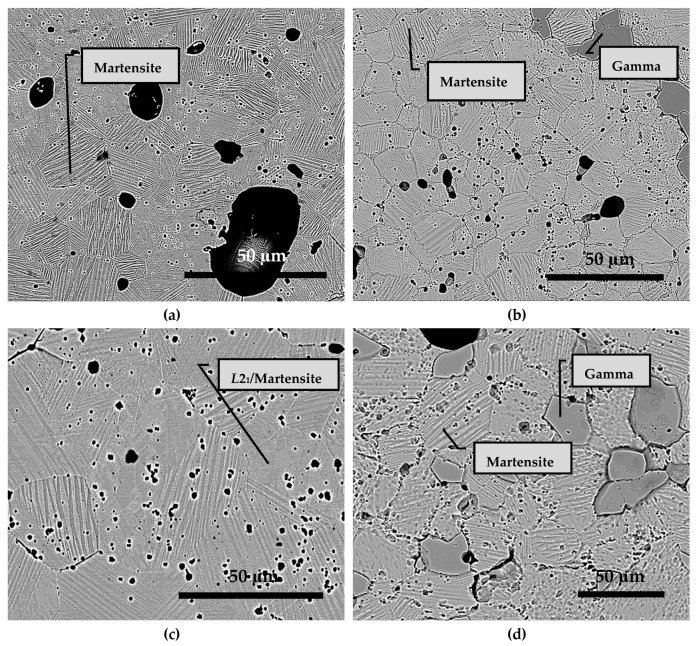
BSE micrographs of Ni_45_Co_5_Mn_40_Sn_10_ alloy: (**a**) S1050a1; (**b**) S1050a2; (**c**) S1050b1; and (**d**) S1050b2.

Figure 8a shows the BSE micrograph of alloy S1050a1 and Figure 8b shows that of alloy S1050a2. Both structures show martensitic grains with “lath” structures clearly seen. The average grain size determined by the intercept method is 13.95 µm and 10.97 µm, respectively. The structures are predominantly single phase, labelled as martensite. However, in S1050a2, additional dark gray regions are seen. S1050b1 has martensitic and non-martensitic regions with an average grain size of 14.57 µm, seen in Figure 8c, while S1050b2, seen in Figure 8d, has a similar microstructure to S1050a2, with an average grain size of 8.28 µm. The decrease in grain size with increasing sintering time does not go with the well-accepted concept that further diffusion will bring about coarsening, i.e., larger grain size. As will be discussed later, this is due to reactions at the grain boundaries, which tend to consume part of the grains, resulting in reduction of grain size, in contradiction to popular belief. Martensitic and non-martensitic regions, along with the dark gray phases, are identified on the micrographs. The compositions of the phases seen in Figure 8 are presented in Table 4. The composition of the dark gray region in all samples shows an increased Co content and only traces of Sn. These dark gray regions with excess Co are identified as γ-phase and the non-martensitic regions are identified as *L*2_1_/martensite, in accordance with literature [20]. The non-martensitic phase is actually the *L*2_1_ austenite, which transforms to martensite upon cooling. From Table 4, it can be seen that the compositions of martensitic and non-martensitic regions are nearly similar. 

**Figure 9:**
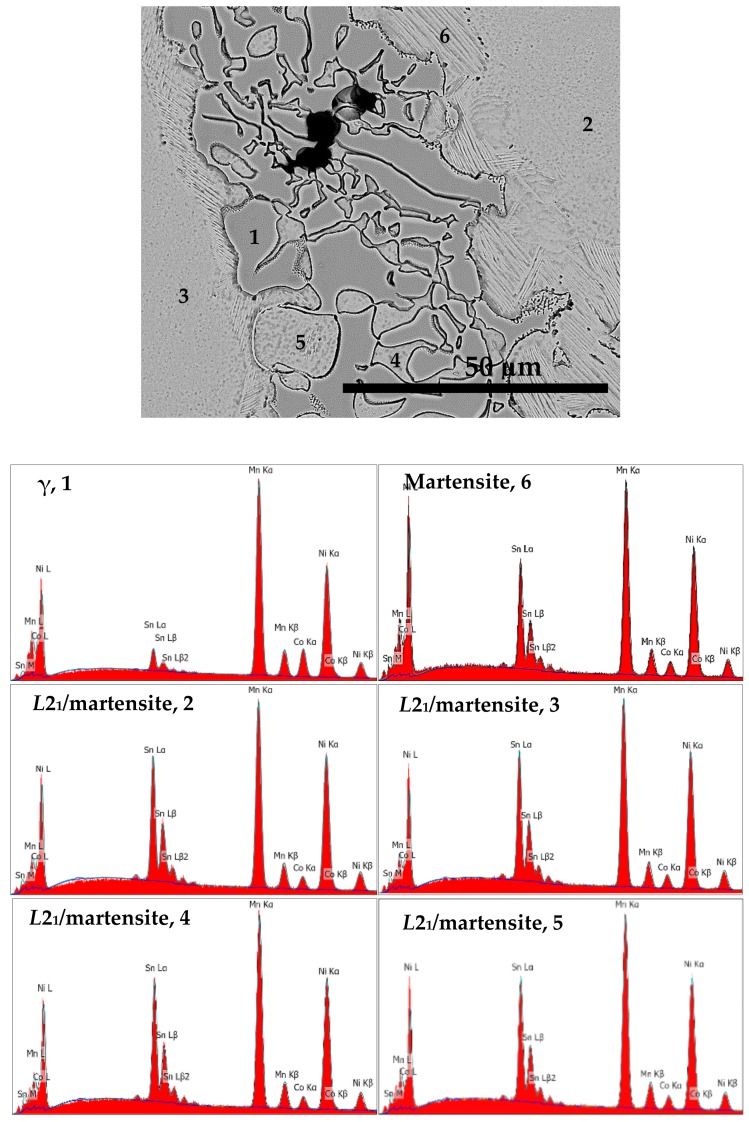
BSE micrograph and EDS spectra of different regions seen in S1050a3.

**Figure 10:**
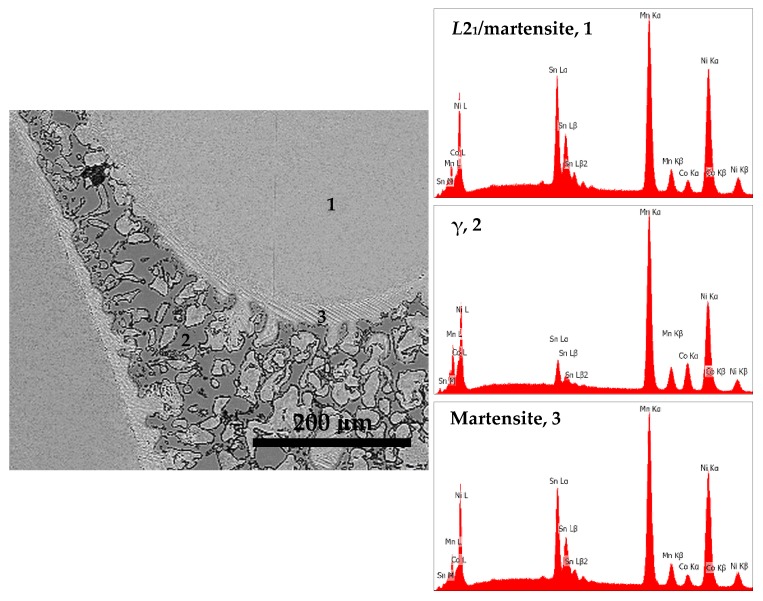
BSE micrograph and EDS spectra of different regions seen in S1050b4.

Figure 9 shows the back scattered electron (BSE) micrograph of S1050a3 alloy and the EDS spectra of different regions in it. Figure 10 shows the micrograph of S1050b4 alloy along with the EDS spectra. The compositions of the phases represented by the regions are included in Table 5. Of identical regions, the compositions are fairly uniform. The microstructures can be seen to resemble a cast structure when melting and solidification takes place. In addition to non-martensitic light gray cells, the inter-cell structure comprises three regions—martensitic structure having a faint lath morphology, predominantly forming at the periphery of the inter-cell region, light gray of a similar feature to that of cells, and dark gray regions having a bulky morphology. It is seen from Figure 9 and Figure 10 that the light gray phase within the inter-cell regions is transformed to bulky dark gray phase. In S1050a2 and S1050b2 samples, sintered for 24 h, the presence of dark gray phase is less and the microstructures resemble a predominantly martensitic structure, as seen in Figure 8b,d respectively. In samples S1050a3 and S1050b4, sintered for 72h and 144 h, the dark gray regions identified as γ-phase earlier are present in large measure, as seen from Figure 9 and Figure 10 and also from Figure 5a,b. The compositions of the phases are included in Table 6. The volume fraction of the γ-phase is more in S1050b4 when compared to S1050a3, which means that increased amounts of γ-phase have stabilized at 144 h of sintering. This is because phases with excess Co stabilize at higher temperatures beyond 950 °C [20]. Thus, with dark gray regions stabilizing in the phase boundaries, relegation of martensite to the boundaries by the light regions occurs because the γ-phase acts as a heat sink for the martensite, which usually forms through a shear and diffusionless mechanism during quenching. The alloys being furnace-cooled underwent an incomplete martensitic transformation with the martensite confined only to the boundary region adjoining the γ-phase. This, together with the formation of the γ-phase, accounts for the absence of distinct martensitic transformation peaks in the DSC graphs.

**Figure 11:**
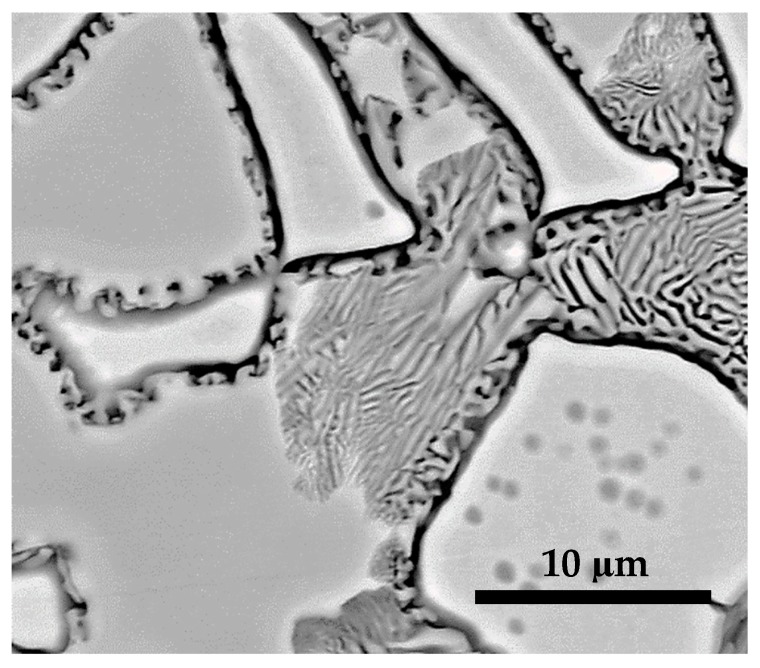
BSE micrographs of S1050a4 higher magnification.

Shown in Figure 11 is the microstructure of S1050a4 at high magnification. This sample is sintered for 144 h. A lamellar structure can be seen within the γ-phase (dark gray regions) at the grain boundaries. The composition of lamellae alternately corresponds to *L*2_1_/martensite (light gray) and γ-phase (dark gray) regions. This is akin to a eutectoid *L*2_1_ and γ-phase precipitating from D0_3_ phase in a solid state [20]. The D0_3_ phase has higher Sn content, while in S1050a4, no such phase is seen. From the microstructure shown in Figure 11, it can be deduced that the non-martensitic light gray region, since D0_3_ is absent, formed the eutectoid structure. Another characteristic of Heusler alloys is that a B2–*L*2_1_ order–disorder transition occurs at around 500–800 °C, however the compositions of the alloys in [20] do not favor such a transition. Therefore, the B2–*L*2_1_ order-disorder transition is not a characteristic in the powder processed alloys being discussed in this work.

**Figure 12:**
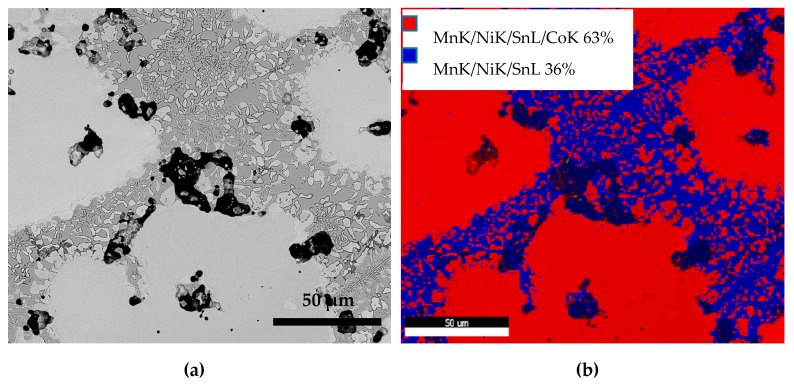
(**a**) BSE micrograph of S1050a3 and (**b**) compositional map of the microstructure.

**Figure 13:**
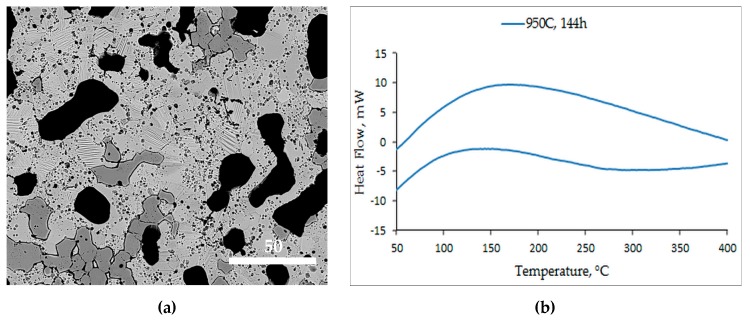
(**a**) BSE micrograph of S950b4 and (**b**) DSC curve of S950b4.

From the composition map of alloy S1050a3 shown in Figure 12b, it can be seen that 63% of the area is light region (red) and 36% contains the dark region (γ-phase), light region, and the eutectoid region (blue). Sample S1050b4, sintered at 144 h, also exhibited around 62% of light region and 37% of light, dark, and eutectoid regions (not shown). Figure 13a shows the BSE micrograph of alloy S1050a3. This also has large light grey regions relegating the martensitic and dark grey regions to and in between the grain boundaries.

The microstructure of alloy S950b4 is shown in Figure 13a. Seen in this microstructure are martensitic, non-martensitic, and dark gray regions, as in any other alloy. The average grain size is 6.95 µm. While the martensitic lath structures are as in S1050a2 and S1050b2, the presence of pores and γ-regions is fairly appreciable. The DSC (Figure 13b) of this alloy does not clearly define any phase transformation, even though the microstructure exhibits martensite. This is because both the structural and compositional homogeneity are not attained in the alloy, in spite of having been sintered for 144 h at 950 °C.

**Figure 14:**
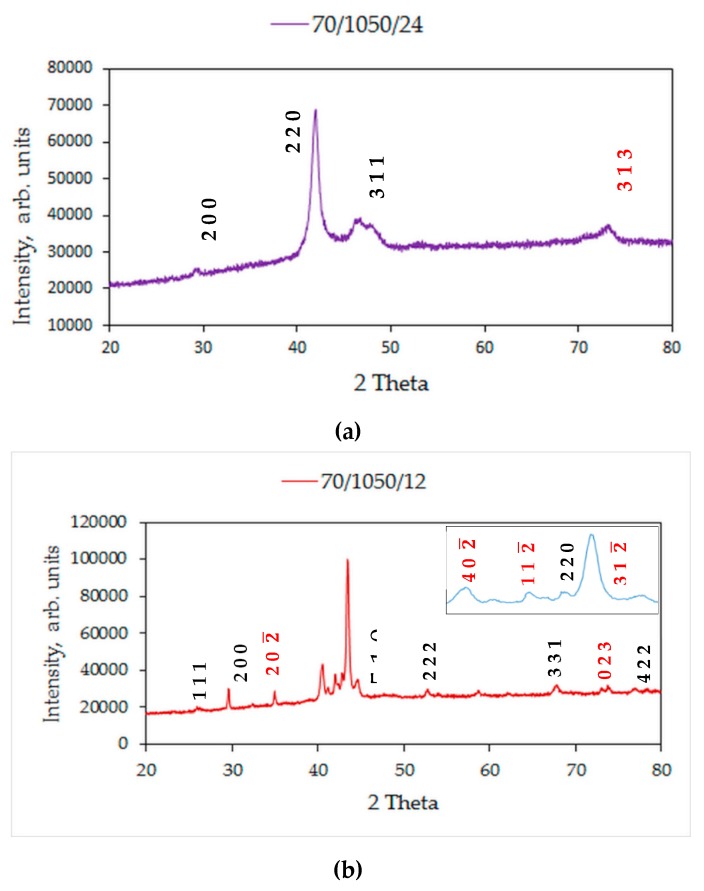
XRD patterns of: (**a**) S1050a2 and (**b**) S1050a1.

**Figure 15:**
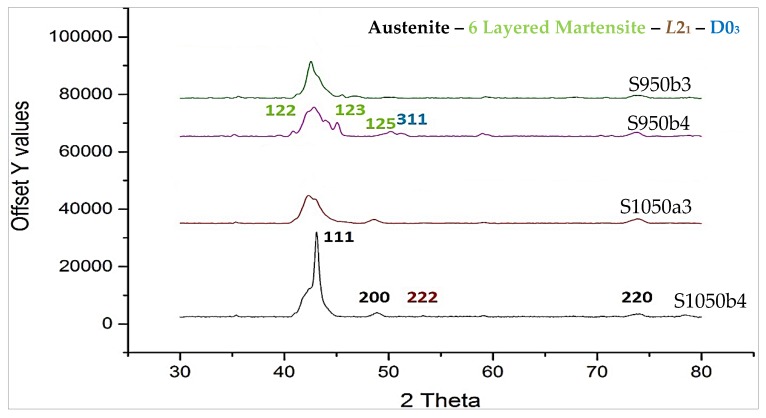
XRD patterns of S1050b4, S1050a3, S950b4, and S950b3.

Figure 14a,b shows the room temperature diffraction patterns of the quaternary samples S1050a2 and S1050a1, sintered at 24 h and 12 h, respectively. The inset in Figure 14b shows 2 theta values from 40 to 45 degrees. The spectrum in Figure 14a was indexed to Heusler *L*2_1_ (space group Fm$\bar{3}$m) in addition to a single peak indexed to monoclinic (6M) phase (space group C12/m1), typed in red. The spectrum in Figure 14b was indexed to Heusler *L*2_1_ (space group Fm$\bar{3}$m, lattice parameter a = 6.0250 Å) and monoclinic (6M) phases (space group C12/m1, lattice parameters: a = 12.2000 Å, b = 4.0550 Å, c = 5.2150 Å, β = 105.03°). Monoclinic reflections are typed in red letters. The coexistence of austenite and martensite in the as-cast Ni_50_Mn_37_Sn_13_ alloys has been reported [37]. The room temperature XRD patterns of alloy S1050b4, S1050a3, S950b4, and S950b3 are shown in Figure 15. They point to the presence of austenite/*L*2_1_ phase (light gray regions in all microstructures) and γ-phase (dark gray), denoted by (220). The γ-phase is seen in all the samples. S950b4 shows the structure is largely six-layered martensite, coexistent with austenite. Additionally, there is a weak reflection, specific to D0_3_ phase, which is absent in samples sintered at 1050 °C.

### 3.4. Magnetization Measurements

**Figure 16:**
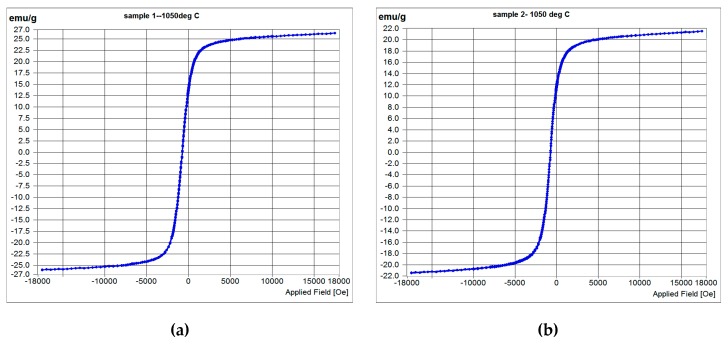
M–H curves of samples: (**a**) S1050b4; (**b**) S1050b3.

Figure 16a,b shows the M–H curves of samples S1050b4 and S1050b3, sintered at 1050 °C for 144 h and 72 h, respectively. The measurements were carried out at room temperature. The magnetic parameters determined from the measurements are shown in Table 7, including S950b4 (M-H curve not shown). All the samples tested for magnetization are soft magnets, seen from the low values of remanence, coercivity, and squareness. 

## 4. Conclusions

The effects of the sintering parameters on the transformation characteristics and microstructure of a Ni_45_Co_5_Mn_40_Sn_10_ alloy synthesized using pressureless powder metallurgy were studied. The findings are summarized as follows:

1. Synthesis of Ni–Co–Mn–Sn Heusler alloys by powder processing using elemental powders is feasible;
2. The effect of compaction pressure on the magnetostructural characteristics is not as significant;
3. A higher sintering temperature of 1050 °C enables adequate sintering to occur, demonstrated by the decrease of the porosity and the increase of hardness;
4. Sintering at 1050 °C results in a predominantly single phase *L*2_1_ structure and a small fraction of γ-phase. At a lesser duration of 12/24 h, the γ-phase is less and at a higher duration of 72/144 h, it stabilizes to around 36% of the alloy composition;
5. At 72/144 h, the *L*2_1_ solidified again into *L*2_1_ and γ-phase in a eutectoid process;
6. A slightly lower process temperature of 950 °C did not ensure adequate diffusion and sintering and, consequently, no martensitic transformation, essential for magnetostructural applications. However, after 144 h, the microstructure had clear martensitic grains with grain refinement. Still, no martensitic transformation was recorded, due to the persistence of inhomogeneity;
7. A processing condition of 1050 °C/24 h is favorable for synthesizing ferromagnetic Ni–Co–Mn–Sn alloys. A secondary thermomechanical procedure is necessary for the elimination of the γ-phase that masks magnetostructural behaviour.

## Figures and Tables

**Table 1:** Particle sizes of different powders.

	Ni (µm)	Mn (µm)	Sn (µm)	Co (µm)
D (v,0.5)	11.52	16.91	19.14	8.05

**Table 2:** Processing conditions of the alloy samples.

Sample ID	Compaction Pressure (MPa)	Holding Time (h)	Temperature (°C)
S950a1	184	12	950
S950a2	184	24	950
S1050a1	184	12	1050
S1050a2	184	24	1050
S950b1	210	12	950
S950b2	210	24	950
S1050b1	210	12	1050
S1050b2	210	24	1050
S1050a3	184	72	1050
S1050a4	184	144	1050
S1050b3	210	72	1050
S1050b4	210	144	1050

**Table 3:** Composition of the alloys obtained from EDS.

Sample ID	Ni (at %)	Mn (at %)	Co (at %)	Sn (at %)	Total
S950a1	44.7	42.8	5.3	7.2	100.0
S950a2	45.8	41.1	5.1	7.9	100.0
S1050a1	44.8	41.8	5.4	8.0	100.0
S1050a2	45.9	39.9	4.6	9.5	100.0
S950b1	45.0	42.2	5.1	7.7	100.0
S950b2	45.2	42.0	4.9	7.8	100.0
S1050b1	44.9	41.0	5.0	8.9	100.0
S1050b2	45.2	40.8	5.1	8.9	100.0
S1050a3	45.8	40.3	5.0	8.9	100.0
S1050a4	44.9	41.6	5.6	7.8	100.0
S1050b3	45.3	40.9	5.9	7.7	100.0
S1050b4	44.6	42.7	5.8	6.9	100.0

**Table 4:** Transformation and hysteresis temperatures obtained from differential scanning calorimetry (DSC).

Sample ID	A_S_ (°C)	A_F_ (°C)	A_P_ (°C)	M_S_ (°C)	M_F_ (°C)	M_P_ (°C)	(A_S_ + M_F_)/2 (°C)	(A_F_ – M_S_) (°C)	ΔH/Jg^−1^	
									Heat	Cool
S950a1	135	–	–	–	103	–	–	–	–	–
S950a2	140	215	175	210	105	156	123	5	20.08	33.75
S1050a1	120	182	160	155	85	125	103	27	26.45	25.60
S1050a2	130	175	157	158	109	135	120	17	12.75	13.89
S950b1	132	–	–	–	–	–	–	–	–	–
S950b2	130	220	170	205	100	155	115	15	19.42	22.66
S1050b1	125	160	148	132	88	112	107	28	22.27	19.56
S1050b2	130	187	174	168	100	140	115	19	32.17	34.29
S1050a3	–	–	–	–	–	–	–	–	–	–
S1050a4	–	–	–	–	–	–	–	–	–	–

**Table 5:** Composition of regions in the microstructures of the S1050a1, S1050a2, S1050b1, and S1050b2.

Sample ID	Ni (at %)	Mn (at %)	Co (at %)	Sn (at %)	Total	In Figure
S1050a1	45.2	40.8	5.1	8.9	100.0	8a: Martensite
S1050a2	45.6	40.4	5.4	8.7	100.0	8b: Martensite
S1050a2	44.9	41.9	11.1	2.1	100.0	8b: γ-phase
S1050b1	45.0	41.7	5.1	8.2	100.0	8c: Martensite
S1050b1	45.5	41.1	4.6	8.8	100.0	8c: Non-martensite
S1050b2	45.0	41.0	5.1	8.9	100.0	8d: Martensite
S1050b2	42.3	45.2	11.3	1.2	100.0	8d: γ-phase

**Table 6:** Composition of regions in the microstructures of the S1050a3 and S1050b4.

Sample ID	Ni (at %)	Mn (at %)	Co (at %)	Sn (at %)	Total	In Figure
S1050a3	46.0	40.2	4.4	9.4	100.0	9: Martensite, 6
S1050a3	44.6	43.7	9.6	2.1	100.0	9: γ-phase, 1
S1050a3	46.9	38.3	3.9	10.9	100.0	9: *L*2_1_/martensite, 2
S1050a3	46.9	38.1	4.0	11.0	100.0	9: *L*2_1_/martensite, 3
S1050a3	45.9	40.0	3.7	10.4	100.0	9: *L*2_1_/martensite, 4
S1050a3	45.7	39.6	4.0	10.7	100.0	9: *L*2_1_/martensite, 5
S1050b4	46.6	39.2	4.5	9.7	100.0	10: *L*2_1_/martensite, 1
S1050b4	40.3	45.4	11.6	2.7	100.0	10: γ-phase, 2
S1050b4	45.9	40.9	4.6	8.6	100.0	10: Martensite, 3

**Table 7:** Magnetic parameters of samples S1050b4 and S1050b3.

Sample	Coercivity, H_C_ Oe	Remanence, M_R_ emu/g	Saturation magnetization, Ms emu/g	Squareness, M_R_/M_S_
S1050b4	4.64	0.09654	26.148	0.532
S1050b3	6.596	0.12719	21.483	0.552
S950b4	7.623	0.1566	12.34	0.593
